# Cytonemes in development

**DOI:** 10.1016/j.gde.2019.06.005

**Published:** 2019-08

**Authors:** Chengting Zhang, Steffen Scholpp

**Affiliations:** Living Systems Institute, Biosciences, College of Life and Environmental Science, University of Exeter, Exeter, EX4 4QD, UK

## Abstract

Cell–cell communication is essential during the development of multicellular organisms. Specialized cell protrusions called cytonemes have been identified to exchange signals between cells that are vital for tissue development. Cytonemes can carry signalling components between distant cells and thus regulate the activity levels of the corresponding signalling pathways across entire tissues. This review summarizes the key findings on the formation and function of cytonemes in tissue development.

**Current Opinion in Genetics and Development** 2019, **58**:25–30

This review comes from a themed issue on **Developmental mechanisms, patterning and evolution**

Edited by **Gáspár Jékely** and **Maria Ina Arnone**

For a complete overview see the Issue and the Editorial

Available online 9th August 2019

**https://doi.org/10.1016/j.gde.2019.06.005**

0959-437X/Crown Copyright © 2019 Published by Elsevier Ltd. All rights reserved.

## Introduction

During the development of multicellular organisms, cells release and receive vital paracrine information to instruct and influence cellular behavior. This intercellular communication controls specific intracellular signalling cascades which allow the recipient cells to grow, to divide or to differentiate. Such an orchestrated cellular behavior is essential for the development of tissues and organs. Specific cell groups, which are active in producing and releasing a variety of signals, are referred to as organizers [1]. The morphogens emitted from organizers instruct the development, form and function of the neighbouring tissues and organs by formation of concentration gradients in the neighbouring tissue. However, the mechanism of morphogen dissemination has been debated. Several ways might allow paracrine signalling to occur, such as dissemination of signalling molecules via diffusion and on exovesicles [2,3]. However, recently, thin and actin-rich membranous protrusions — signalling cytonemes — have emerged as key players in mobilizing morphogens in a tissue during embryonic development [4, 5, 6]. Cytonemes have been shown to transport essential components of signalling pathways such as BMP, Fgf, Shh, and Wnt between cells. These cellular extensions present some unique and characteristic features in comparison to other suggested transport mechanisms: Cytonemes are highly dynamic and can form and retract within minutes. They can extend over hundreds of micrometres to contact distant cells. Their emergence is precisely controlled by the cytoneme-producing cell and the extracellular space through which they traverse. In addition, the targeted cell – including the subcellular region on the target-cell membrane to which the cytonemes connect – is carefully chosen. Importantly, cytonemal transport allows a high degree of organization in exchanging essential information during tissue development. However, this communication route requires a complex molecular mechanism to regulate all the attributes mentioned above.

In this review, we will elucidate recent advances in our understanding of the presentation of signals through cytonemes to which refer to as cytoneme-mediated transport. Specifically, we will describe the requirement of cytonemal transport for signalling components of the BMP, Wnt and Shh families. We will further examine which molecular mechanisms underlie the behavior of cytonemes. Finally, we will discuss the consequences of cytoneme-based transport on gradient formation.

## Cytonemes operate in various signalling networks

**Figure 1:**
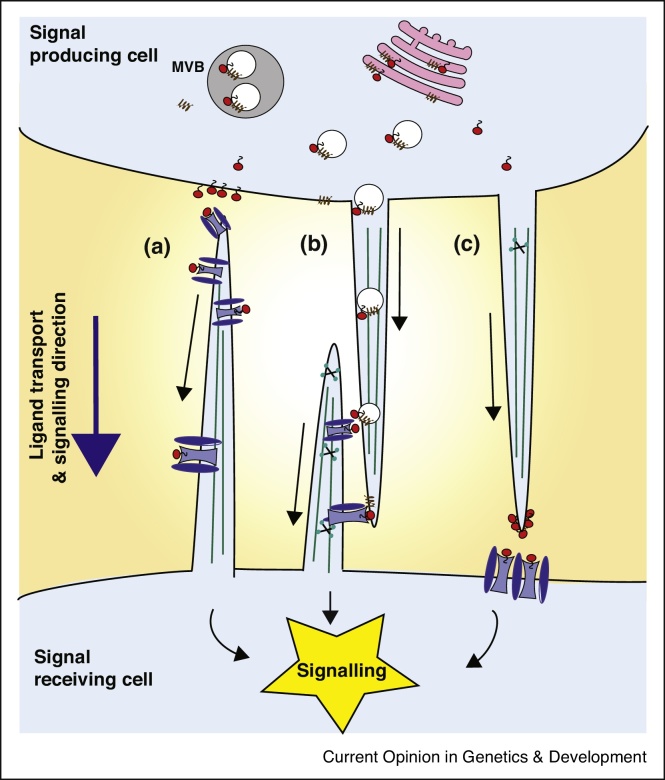
Cytoneme-based transport of signalling components. **(a)** Cytonemes can be loaded with receptors and be extend towards the signal producing cell and Fgf trafficking in flies serves as an example. **(b)** Ligands and receptors can be loaded on cytonemes and inter-cytonemal contacts are established as seen for Hh signalling. **(c)** The ligands can be mobilized on cytonemes and transported to the receiving cells and, indeed, Wnt proteins are tranported on cytonemes in zebrafish development.

Cytonemes have been first described in the Drosophila wing imaginal disc [7^•^]. These signalling filopodia are thin, actin-rich protrusions and respond to signals from morphogen-producing centres. Decapentaplegic (Dpp), which belongs to the BMP family, is required for patterning the wing disc [8,9]. Dpp is expressed at the anteroposterior boundary and signals to neighbouring cells of the wing disc epithelium. Dpp-receiving cells from the wing imaginal disc package the Dpp receptor Thickveins (Tkv) on apical cytonemes and direct these to the Dpp source (Figure 1a) [10,11]. Abrogation of cytonemes restricts the range of Dpp signalling and thus causes reduced growth.

In addition to Dpp, Fgf signalling is also essential in *Drosophila* embryogenesis with an essential role for the development and maintenance of the tracheal system [12,13]. Indeed, cells of the air sac primordium require Dpp and Fgf to regulate proliferation and differentiation. Similar to the situation in the wing imaginal disc, air sac primordium cells of developing trachea in the wing disc load the Dpp receptor Tkv or the Fgf receptor breathless (Btl) on tracheal cytonemes, which orient towards the morphogen sources in the wing disc [14^•^,^15^]. However, cytonemes are only loaded with either Tkv or Btl, indicating high specificity. In turn, Fgf signalling induces the formation of longer signalling filopodia to allow a more efficient uptake of the ligand from the producing cells [12,14^•^]. Another example is the eye imaginal disc: cells in the eye disc make cytonemes that respond to Egf/Spitz. A common feature of these signalling systems is that the specific ligands are hydrophilic and the transmembrane spanning receptors are loaded onto cytonemes. Although, the nature of the ligand would allow a diffusion-based propagation, the receptors pick up the ligand directly at the source cell. We can assume that such a mechanism allows precisely controlled and directed long-range signalling.

During Hedgehog (Hh) signalling and Wnt signalling, both the morphogen ligands and their receptors can be distributed by cytonemes [16]. These signalling systems require the distribution of lipid-modified, hydrophobic ligands, which presumably complicates diffusion-based transport. The Hh protein is covalently modified by two lipid moieties, cholesterol and palmitate, and their hydrophobic properties are thought to govern cellular release. In *Drosophila*, Hh is produced in the posterior wing compartment. After lipid-modification, Hh is routed to the basal side of the wing epithelium, where it is released and forms a signalling gradient [17]. Hedgehog morphogen is transported via vesicles along cytonemes, which act as conduits for morphogen movement (Figure 1b) [18]. In addition to the signal-producing cells, cytonemes also protrude from Hh-receiving cells [19^•^,20^••^]. The canonical Hh receptor Patched is localized on these cellular protrusions and Hh reception takes place at membrane contact sites between Hh-sending and Hh-receiving cytonemes. The vertebrate homologue Sonic Hedgehog (Shh) is expressed in the polarizing region (or zone of polarizing activity), a small group of mesenchymal cells at the posterior margin of the vertebrate limb bud. In the chick limb, Shh is transported to the responding cells through cytonemes [21]. Similar to the situation in *Drosophila*, the Shh receptor, BOC, can also be found on cytonemes of Shh-receiving cells in chicken, which expands the signalling range to several hundreds of μm in the developing chick limb anlage.

Finally, Wnt signalling is critical for cell proliferation and differentiation during embryogenesis. Wnt ligands are also lipid-modified. For example, molecular analysis of Wnt3a revealed that the serine 209 is modified with a monounsaturated fatty acid, a palmitoleic acid [22]. Cytonemes are also fundamental for transporting Wnt signals. Cytonemal transport of Wnt8a is essential during neural plate patterning during zebrafish gastrulation [23^••^]. Wnt is loaded on cytonemes and can be found at the cytonemal tip (Figure 1c). Wnt cytonemes have also been detected in mouse intestinal crypts in which myofibroblasts send Wnt signals via cytonemes to the intestinal crypt cells [24^••^]. In chick, there is evidence that also the Wnt receptor Frizzled7 (Fzd7) is required for somite formation and Fzd7 puncta could be detected on cytonemes emitting from the ectodermal layer [25]. These findings are similar to observations in *Drosophila,* in which cytonemes containing Fzd receptors extend from myofibroblasts to pick up Wg signal from the wing disc [26]. Therefore, in Wnt signalling there is evidence that both the ligand as well as the receptor can be loaded on cytonemes to exchange Wnt signals [27].

Besides their function in development, cytonemes also play an important role in tissue homeostasis. Cytoneme-based transport seems to be crucial in cancer as Wnt cytonemes can regulate proliferation in gastric cancer cells [24^••^]. Complementary to this, the inhibition of essential cytoneme regulators such as diaphanous and capricious inhibits tumor growth and restores the apical basal polarity to tumour cells in *Drosophila* [28]. These results indicate that cytoneme-mediated signalling can be crucial for tumour growth and malignancy. During zebrafish colour pattern formation, actin-containing filopodia from macrophages contact neighbouring xanthophores to relay long-range signalling between non-immune cells [29^•^]. In summary, we find that the paracrine activation of several signalling pathways relies on cytonemes. This raises the question whether filopodia-mediated exchange is the advancement of juxtacrine signalling between directly neighbouring cells in expanding tissues and organisms. Furthermore, it seems crucial to analyse the molecular nature of the ligands to predict which signalling components are loaded on cytonemes and if an anterograde and/or retrograde transport of the ligand can be expected.

## Formation and function of cytonemes

Because of the important role of specialized filopodia during signal transport in development, the focus in cytoneme biology has shifted towards deciphering the molecular mechanisms which underlie their formation, in particular the length and the number of filopodia, which affects downstream signalling. In several mathematical models, these parameters have been highlighted as crucial to determining signalling range and potentially the morphogen gradient [30,31]. In general, cytoneme emergence can be controlled by altering the actin cytoskeleton. Cytonemes share many characteristics with filopodia. Therefore, blockage of filopodia regulators are often used to interfere with cytonemes. However, the identification of cytoneme-specific regulators remains in its infancy. Recent experiments in zebrafish have started to shed light on the underlying mechanism. Wnt cytonemes are regulated by autocrine Wnt/Planar Cell Polarity (PCP) signalling [24^••^]. Wnt8a binds to the receptor tyrosine kinase Ror2 to activate the filopodia nucleation machinery including RhoA-dependent signalling. Ror2 is crucial for the induction of cytonemes, thus, enhanced Ror2 signalling leads to an increase in cytoneme number. Furthermore, the downstream regulators of the PCP pathway, Cdc42 and N-Wasp control length and branching behavior of Wnt cytonemes [23^••^]. In the mouse intestinal crypt, filopodia formation can be stimulated through activation of the stem cell markers Lgr4/5, therefore, it is tempting to speculate that these filopodia carry signalling factors [32,33]. In conclusion, it seems that signalling pathway-specific components such as co-receptors are required to regulate the filopodia nucleation machinery. This assumption could also explain the specificity of cytonemes for individual signalling systems [34]. The identification of these factors for the other cytoneme-dependent pathways described above will be an important aim in cytoneme biology in the future.

## Cytonemes and morphogenetic fields

**Figure 2:**
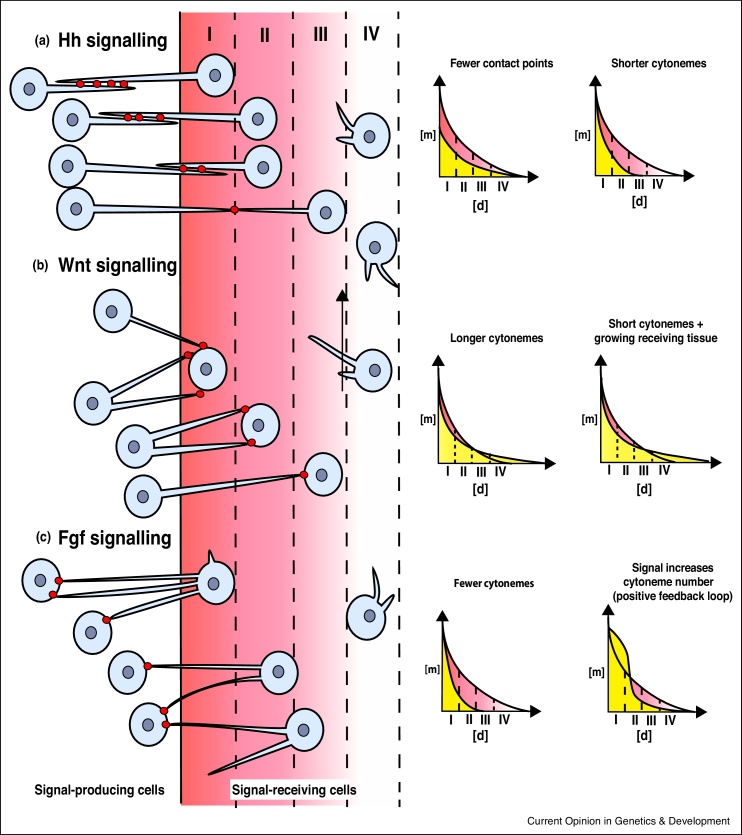
Morphogentic field formation and cytonemes. **(a)** The Hh gradient is influenced by number of cytonemal contact points between cytonemes. **(b)** The Wnt gradient in the neural plate in zebrafish is regulated by the number of cytoneme contact sites. In addition, the expansion of the target tissue ensures long-range signalling. **(c)** The Fgf signalling gradient in air sac primordium cells in Drosophila is determined by the number of cytonemes and a positive-feedback loop regulation as Fgf signalling enhances cytoneme formation. The red graphs illustrate the intial gradient, whereas the yellow curves illustrate the gradient after the indicated alterations in cytoneme emergence.

Many theories invoked the concept of *morphogens* – chemical signalling molecules capable of directing the behavior and differentiation of cells. The seminal model of Turing proposed that a system of reacting and diffusing molecules could form a spatially periodic pattern [35]. Subsequently, the French-flag model of Wolpert proposed that a non-uniform distribution of a morphogen could pattern a tissue via distinct differentiation paths being followed by cells according to the morphogen level [36]. However, these models are based on the assumption that morphogens are disseminated via diffusion. How could morphogen gradients form in light of the recent advances in contact-mediated morphogen transport? Key morphogens in development are the Hh and Fgf proteins, which form morphogen gradients to influence tissue patterning. Hedgehog is transported from the posterior compartment to the anterior compartment cells in the *Drosophila* wing disc. Cells from both compartments form cytonemes and the two sets of cytonemes make contact through the basal side of the epithelium. Hh is loaded on exosomes and transported through cytonemes, which act as conduits [18,37]. The distance to the source and the steepness of the gradient is influenced by the length of the cytonemes and by the number of contact points along individual cytonemes (Figure 2a) [19^•^].

A fundamentally different way to establish a morphogenetic gradient has been described in zebrafish neural plate patterning [38]. In this context, the initial range of the signalling gradient is set by the length of the cytonemes. However, during development of the neural plate, the tissue extends from 50 μm to 600 μm, although the Wnt source is kept constant. Therefore, Wnt-receiving tissue is expanding and cells are continuously moving out of the cytonemal area of influence. Consequently, the lengths of the cytonemes and tissue movement – that is, expansion on the neural plate – contribute to the range of the gradient (Figure 2b). The steepness, in contrast, is determined by the frequency of contacts by cytonemes, as Wnt is exchanged mainly on the tip of cytonemes and therefore one cytoneme equals one event of signal activation.

A third option was put forward to explain the establishment of the Fgf gradient in the Drosophila air sac primordium. In the tracheal system, airway cells load the Fgf receptor Btl on cytonemes to pick up Fgf ligand from the wing imaginal disc [14^•^]. The Fgf gradient in the airway tissue is created because airway cells near Fgf producing cells generate more cytonemes, and therefore collect more Fgf ligand compared to more distant cells (Figure 2c). The graded number of cytonemes regulate, therefore, the steepness of the Fgf morphogen gradient. Interestingly, the Fgf signal induces the formation of cytonemes. This positive feedback loop increases the steepness of the slope over time. In summary, there are several ways that suggest that morphogen gradients can be formed if cells communicate via cytonemes. This further suggests that we need to develop novel mathematical models to explain the idea of gradient formation according to Turing and Wolpert however based on cytonemal distribution of the morphogenetic signal.

## Conclusion

Since the initial observation of cytonemes 20 years ago, many reports have highlighted the importance of cytonemes in morphogenetic systems. Cytonemal transport of signalling components have specific advantages compared to a diffusion-based ligand transport as illustrated in this review. However, cytoneme biology is still in its infancy. A striking feature of cytonemes is the possibility of a two-way communication: To date, research focuses on cytoneme-mediated signal transport and the activation of signalling cascades in the receiver cell. However, it may also signal backwards to the producing cell, but it is unknown how such retrograde signalling is conveyed. Furthermore, the physical properties of a cytoneme contact site and its influence on signalling needs to be explored in the future. For example, the underlying signal sensing and physical force application through cellular protrusions remains to be investigated. However, these questions were outshone by one of the most exciting findings, that essential factors of neuronal signalling at synapses have been identified at cytoneme contact sites [39^••^]. Manipulation of calcium signalling and alteration of the activity of glutamate receptors such as GluRII influence cytoneme-mediated Dpp signalling in Drosophila. These observations suggest that cytoneme contact sites share common features with a neuronal synapse and the future will tell if cytoneme contact sites and synapses share a common origin.

Considering the multifunctional facets of cytoneme-mediated signalling much further research is needed elucidating the aspects on the molecular, cellular and tissue levels of this exciting transport mechanism before intercellular communication is fully understood.

## Conflict of interest statement

Nothing declared.

## References and recommended reading

Papers of particular interest, published within the period of review, have been highlighted as:

• of special interest
•• of outstanding interest

## References

[1] Arias A.M., Steventon B. (2018). On the nature and function of organizers. Development.

[2] Müller P., Rogers K.W., Shuizi R.Y., Brand M., Schier A.F. (2013). Morphogen transport. Development.

[3] Akiyama T., Gibson M.C. (2015). Morphogen transport: theoretical and experimental controversies. Wiley Interdiscip Rev Dev Biol.

[4] Gradilla A.-C., Guerrero I. (2013). Hedgehog on the move: a precise spatial control of Hedgehog dispersion shapes the gradient. Curr Opin Genet Dev.

[5] Roy S., Kornberg T.B. (2015). Paracrine signaling mediated at cell–cell contacts. BioEssays.

[6] Mattes B., Scholpp S. (2018). Emerging role of contact-mediated cell communication in tissue development and diseases. Histochem Cell Biol.

[7•] Ramírez-Weber F.-A., Kornberg T.B. (1999). Cytonemes: cellular processes that project to the principal signaling center in *Drosophila* imaginal discs. Cell.

[8] Kingsley D.M. (1994). The TGF-beta superfamily: new members, new receptors, and new genetic tests of function in different organisms. Genes Dev.

[9] Raftery L., Sanicola M., Blackman R., Gelbart W. (1991). The relationship of decapentaplegic and engrailed expression in *Drosophila* imaginal disks: do these genes mark the anterior-posterior compartment boundary?. Development.

[10] Tanimoto H., Itoh S., ten Dijke P., Tabata T. (2000). Hedgehog creates a gradient of DPP activity in *Drosophila* wing imaginal discs. Mol Cell.

[11] Hsiung F., Ramirez-Weber F.-A., Iwaki D.D., Kornberg T.B. (2005). Dependence of *Drosophila* wing imaginal disc cytonemes on Decapentaplegic. Nature.

[12] Sato M., Kornberg T.B. (2002). FGF is an essential mitogen and chemoattractant for the air sacs of the *Drosophila* tracheal system. Dev Cell.

[13] Franch-Marro X., Cruz J., Martín D. (2018). Dual role of Bnl/Fgf signaling in proliferation and endoreplication of *Drosophila* tracheal adult progenitor cells. BioRxiv.

[14•] Du L., Sohr A., Yan G., Roy S. (2018). Feedback regulation of cytoneme-mediated transport shapes a tissue-specific FGF morphogen gradient. eLife.

[15] Roy S., Hsiung F., Kornberg T.B. (2011). Specificity of *Drosophila* cytonemes for distinct signaling pathways. Science.

[16] Rojas-Ríos P., Guerrero I., González-Reyes A. (2012). Cytoneme-mediated delivery of hedgehog regulates the expression of bone morphogenetic proteins to maintain germline stem cells in *Drosophila*. PLoS Biol.

[17] Callejo A., Bilioni A., Mollica E., Gorfinkiel N., Andrés G., Ibáñez C., Torroja C., Doglio L., Sierra J., Guerrero I. (2011). Dispatched mediates Hedgehog basolateral release to form the long-range morphogenetic gradient in the Drosophila wing disk epithelium. Proc Natl Acad Sci U S A.

[18] Gradilla A.-C., González E., Seijo I., Andrés G., Bischoff M., González-Mendez L., Sánchez V., Callejo A., Ibáñez C., Guerra M. (2014). Exosomes as Hedgehog carriers in cytoneme-mediated transport and secretion. Nat Commun.

[19•] González-Méndez L., Seijo-Barandiarán I., Guerrero I. (2017). Cytoneme-mediated cell-cell contacts for Hedgehog reception. eLife.

[20••] Chen W., Huang H., Hatori R., Kornberg T.B. (2017). Essential basal cytonemes take up Hedgehog in the *Drosophila* wing imaginal disc. Development.

[21] Sanders T.A., Llagostera E., Barna M. (2013). Specialized filopodia direct long-range transport of SHH during vertebrate tissue patterning. Nature.

[22] Takada R., Satomi Y., Kurata T., Ueno N., Norioka S., Kondoh H., Takao T., Takada S. (2006). Monounsaturated fatty acid modification of Wnt protein: its role in Wnt secretion. Dev Cell.

[23••] Stanganello E., Hagemann A.I., Mattes B., Sinner C., Meyen D., Weber S., Schug A., Raz E., Scholpp S. (2015). Filopodia-based Wnt transport during vertebrate tissue patterning. Nat Commun.

[24••] Mattes B., Dang Y., Greicius G., Kaufmann L.T., Prunsche B., Rosenbauer J., Stegmaier J., Mikut R., Özbek S., Nienhaus G.U. (2018). Wnt/PCP controls spreading of Wnt/β-catenin signals by cytonemes in vertebrates. eLife.

[25] Sagar, Pröls F., Wiegreffe C., Scaal M. (2015). Communication between distant epithelial cells by filopodia-like protrusions during embryonic development. Development.

[26] Huang H., Kornberg T.B. (2015). Myoblast cytonemes mediate Wg signaling from the wing imaginal disc and Delta-Notch signaling to the air sac primordium. eLife.

[27] Gradilla A.-C., Sanchez-Hernandez D., Brunt L., Scholpp S. (2018). From top to bottom: cell polarity in Hedgehog and Wnt trafficking. BMC Biol.

[28] Fereres S., Hatori R., Hatori M., Kornberg T.B. (2018). Cytoneme-mediated signaling essential for tumorigenesis. bioRxiv.

[29•] Eom D.S., Parichy D.M. (2017). A macrophage relay for long-distance signaling during postembryonic tissue remodeling. Science.

[30] Verbeni M., Sánchez O., Mollica E., Siegl-Cachedenier I., Carleton A., Guerrero I., i Altaba A.R., Soler J. (2013). Morphogenetic action through flux-limited spreading. Phys Life Rev.

[31] Vasilopoulos G., Painter K.J. (2016). Pattern formation in discrete cell tissues under long range filopodia-based direct cell to cell contact. Math Biosci.

[32] Snyder J.C., Rochelle L.K., Marion S., Lyerly H.K., Barak L.S., Caron M.G. (2015). Lgr4 and Lgr5 drive the formation of long actin-rich cytoneme-like membrane protrusions. J Cell Sci.

[33] Moti N., Yu J., Boncompain G., Perez F., Virshup D.M. (2018). Visualizing Wnt secretion from endoplasmic reticulum to filopodia. bioRxiv.

[34] Huang H., Kornberg T.B. (2016). Cells must express components of the planar cell polarity system and extracellular matrix to support cytonemes. eLife.

[35] Turing A.M. (1952). The chemical basis of morphogenesis. Philos Trans R Soc Lond B Biol Sci.

[36] Wolpert L. (1968). The French flag problem: a contribution to the discussion on pattern development and regulation. Towards Theor Biol.

[37] Bischoff M., Gradilla A.-C., Seijo I., Andrés G., Rodríguez-Navas C., González-Méndez L., Guerrero I. (2013). Cytonemes are required for the establishment of a normal Hedgehog morphogen gradient in *Drosophila* epithelia. Nat Cell Biol.

[38] Stanganello E., Scholpp S. (2016). Role of cytonemes in Wnt transport. J Cell Sci.

[39••] Huang H., Liu S., Kornberg T.B. (2019). Glutamate signaling at cytoneme synapses. Science.

